# The regulation of Xrp1 expression by uORFs and main ORF sequences and its function in *Drosophila* disease models

**DOI:** 10.1371/journal.pgen.1012203

**Published:** 2026-06-10

**Authors:** Hidetaka Katow, Thao Nguyen, Sarah Hyunsoh Park, Hyung Don Ryoo

**Affiliations:** Department of Cell Biology, NYU Grossman School of Medicine, New York, New York, United States of America; Shanghai Institute of Organic Chemistry, Chinese Academy of Sciences, CHINA

## Abstract

The Integrated Stress Response (ISR) mediates cellular adaptation to endoplasmic reticulum (ER) stress, amino acid deprivation, and mitochondrial dysfunction. The ISR regulates gene expression in part by preferentially translating the transcription factor ATF4, a process regulated by upstream open reading frames (uORFs) in its 5’ leader. In *Drosophila,* Xrp1 is another transcription factor induced during the ISR, but the precise underlying mechanism remains unclear. Here, we report that Xrp1 induction in response to ER stress is regulated by both its uORFs and the main ORF sequence. *Xrp1* has seven splice isoforms, and the two predominant transcripts expressed in eye imaginal discs contain uORFs. Expressing the ER stress-imposing *ninaE*^*G69D*^ transgene in this tissue induced *Xrp1* expression without significantly changing the *Xrp1* splice isoform composition. The uORF-containing 5’ leaders, particularly the AUG codon of the second uORF, inhibited DsRed expression when placed upstream of the reporter. Unlike ATF4, the uORF-containing 5’ leader alone was insufficient to mediate the main ORF induction, but Xrp1 induction occurred in *ninaE*^*G69D*^-expressing discs when Xrp1’s 5’ leader and the main ORF sequence were both present. Functionally, *Xrp1* was required to maintain the integrity of *Drosophila* photoreceptors exposed to constant light. In a different disease model, *parkin* mutants activated *Xrp1* target gene expression in specific tissues and *Xrp1* loss enhanced the viability of *parkin* mutant flies during adult eclosion. These results provide molecular and pathological insights into *Xrp1* regulation and function in disease models.

## Author summary

Cells respond to stress through a conserved pathway known as the Integrated Stress Response (ISR), which modulates cellular homeostasis. A key feature of this response is the production of specific transcription factors that regulate stress-adaptive genes. While the mechanism regulating the canonical transcription factor ATF4 is well understood, how other stress-induced transcription factors are controlled remains unclear. Here, we investigate the regulation of Xrp1, a stress-responsive transcription factor in *Drosophila*. We show that, unlike ATF4, Xrp1 induction during stress involves not only regulatory sequences in its 5’ leader but also its main open reading frame (ORF) sequence. We further find that the relative ratio of *Xrp1* isoforms is not significantly altered during stress, suggesting that its regulation occurs primarily at the post-transcriptional level. Importantly, we demonstrate that *Xrp1* has functional roles in disease models. It is required for the survival of light-stressed photoreceptor cells and influences outcomes in *parkin* mutants. Our work reveals the mechanism of *Xrp1* regulation and highlights its importance in disease-related processes.

## Introduction

Eukaryotic cells have evolved a range of stress response pathways that can promote cellular adaptation to physiological or environmental stress, or in certain cases, mediate cell death and dysfunction. One such pathway is the Integrated Stress Response (ISR), which is initiated by stress-activated eIF2α kinases. The ISR is activated by diverse forms of cellular stress such as amino acid deprivation, endoplasmic reticulum (ER) stress, and mitochondrial dysfunction, and affects the course of many neurodegenerative diseases, metabolic disorders, and cancer [1,2].

*Drosophila* disease models are actively utilized to study degenerative phenotypes associated with the ISR. For example, the impairment of major ER stress response mediators leads to retinal degeneration in *Drosophila* [3–5]. Constant light exposure is a source of ER stress in photoreceptors and a failure to properly regulate ER chaperones can cause light-dependent disintegration of photoreceptors [6]. In addition, ISR induction in a model of Retinitis Pigmentosa has been an active area of research [7,8]. Specifically, autosomal dominant Retinitis Pigmentosa (adRP) in humans is most frequently associated with the rhodopsin P23H allele that imposes ER stress to activate the ISR [9]. Similarly, the *Drosophila ninaE*^*G69D*^ allele encodes a mutant Rhodopsin-1 (Rh-1) protein that imposes stress in the ER and causes age-related retinal degeneration [10,11]. *Parkin* is another disease-associated gene required to clear damaged mitochondria through autophagy [12]. The loss of *parkin* strongly induces the ISR in many *Drosophila* tissues, impacting the viability of the organism [5,13].

Once the ISR is initiated by stress-activated kinases that phosphorylate eIF2α, specific transcription factors undergo preferential translation. The well-established ISR transcription factors include yeast GCN4 and metazoan ATF4 [14–19]. These transcription factors contain regulatory upstream open reading frames (uORFs) that inhibit the main ORF translation in unstressed cells, while allowing the preferential translation of the main ORF in response to eIF2α phosphorylation [15]. Upon induction, these factors induce downstream target genes involved in amino acid biosynthesis, protein folding in the ER, and antioxidant response [1,20,21].

More recent studies have linked the ISR with another *Drosophila* bZIP transcription factor, Xrp1. This transcription factor has been extensively examined in *Drosophila Minute* mutants with ribosomal protein (Rp) gene haploinsufficiency [22–24]. In the presence of neighboring wild type cells, these Minute cells are eliminated through apoptosis in a process known as cell competition [25,26]. In this model, *Xrp1* transcripts undergo RpS12-mediated alternative splicing, thereby producing a shorter protein isoform that promotes the death of Minute cells [22,27–30]. In the cell competition model, *Xrp1* lies genetically upstream of eIF2α phosphorylation, as the phospho-eIF2α signals in the Minute cells are suppressed in the *Xrp1* loss-of-function background. Conversely, overexpression of *Xrp1* induces eIF2α phosphorylation, which activates a feed-forward loop to further enhance Xrp1 expression [31–35].

We had previously examined Xrp1 in a distinct experimental setup in which ER stress is imposed by expressing *ninaE*^*G69D*^ in eye imaginal discs using *GMR-Gal4* [36]. In this model, Xrp1 induction occurs downstream of eIF2α phosphorylation, in contrast to its role in *Minute* mutants. Specifically, Xrp1 induction was abolished by the loss of the eIF2α kinase PERK (Pancreatic eIF2α Kinase), whereas overexpression of PERK was sufficient to induce Xrp1 protein in eye imaginal discs [36]. One possible mechanism of Xrp1 induction in response to ER stress is through putative uORFs analogous to those found in ATF4 [36]. However, there are conflicting reports as to whether these uORFs simply inhibit the main ORF translation, or whether they also mediate the induction of Xrp1 in response to stress [29,37].

In this study, we report the mechanisms underlying Xrp1 induction in response to ER stress in eye imaginal discs and demonstrate its functional role in *Drosophila* models of Retinitis Pigmentosa and Parkinson’s Disease. First, we find that the uORF-containing *Xrp1* transcripts are the predominant isoforms expressed in eye imaginal discs, and ER stress does not alter the isoform composition. We further show that the second uORF inhibits the main ORF translation. Xrp1 reporter induction in response to ER stress in eye imaginal discs requires its 5’ leader as well as the main ORF sequence. We further show Xrp1’s roles in degenerative phenotypes. Specifically, *Xrp1* loss-of-function mutants show age-related retinal degeneration. In addition, the *Drosophila parkin* mutants show Xrp1 target reporter induction in several tissues, and *Xrp1* loss enhances the viability of *parkin* mutants after adult eclosion. Together, these findings clarify how Xrp1 mediates ISR induction and establish its physiological relevance in disease models.

## Results

### *Xrp1* transcript composition does not change significantly in response to ER stress caused by *ninaE*^*G69D*^ expression in eye discs

**Fig 1:**
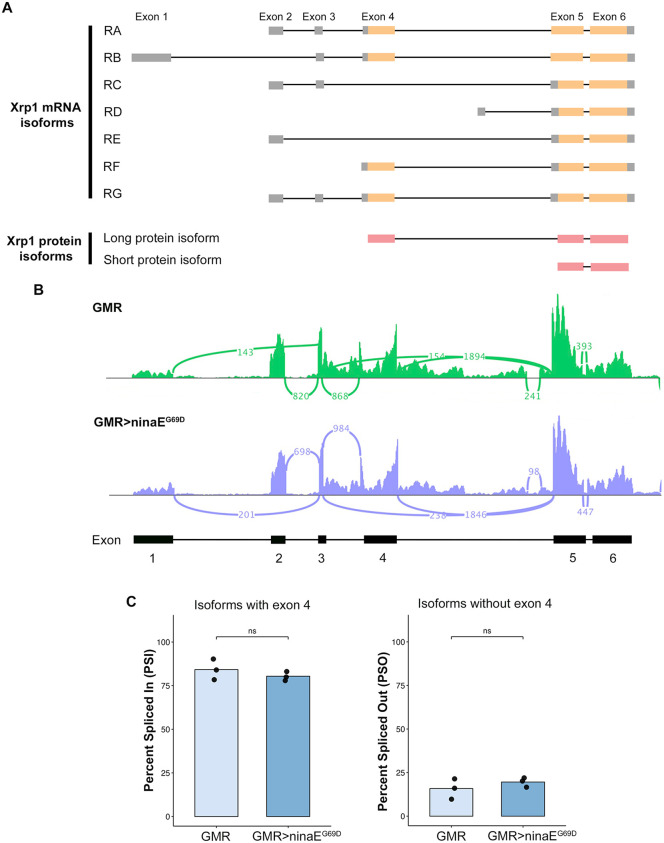
Changes in Xrp1 splice isoform composition in response to ER stress. **(A)** Schematic of *Xrp1* mRNA splice isoforms. Transcripts RA, RB, RF, and RG encode the long protein isoform (Xrp1^long^), whereas RC, RD, and RE encode the short protein isoform (Xrp1^short^). Note that the inclusion of exon 4 distinguishes the long from the short isoform. **(B)** Sashimi plots of RNA-seq reads at the *Xrp1* locus from eye imaginal discs under control *(GMR>EGFP-KASH)* and ER stress (*GMR>ninaE*^*G69D*^ *+ EGFP-KASH*) conditions. Exon coverage is shown along the gene model, and numbers indicate exon-exon junction read counts. Junction usage is comparable between conditions. **(C)** Quantification of exon 4 inclusion and exclusion in *Xrp1* transcripts. Percent Spliced In (PSI; left) and Percent Spliced Out (PSO; right) values are shown for control (*GMR>EGFP-KASH*) and ER stress conditions (*GMR>ninaE*^*G69D*^ *+ EGFP-KASH*). Bars represent mean ± SEM, with individual biological replicates (n = 3 per genotype) overlaid. No significant difference was observed between conditions (two-tailed Student’s t-test; ns, not significant).

The *Xrp1* locus has seven different mRNA splice isoforms (Fig 1A). Transcripts RA, RB, RF, and RG encode the Xrp1 long protein isoform (Xrp1^long^), whereas RC, RD, RE encode the Xrp1 short protein isoform (Xrp1^short^). The presence of the exon 4-encoded sequence in the mRNA distinguishes the long from the short isoform. Previous work has reported that cells with Rp mutations have an increased amount of the Xrp1^short^ isoforms to promote their elimination during cell competition [29,30]. This raises the possibility that stress can regulate Xrp1 activity through alternative splicing.

To test if Xrp1 isoform composition changes in our ER stress model, we analyzed our previously published RNA-seq data from the *Drosophila* eye imaginal discs expressing the Rhodopsin-1 mutant allele, *ninaE*^*G69D*^, which strongly imposes ER stress when expressed in the larval eye imaginal discs. In this dataset, the nuclear membrane-associated EGFP-KASH was co-expressed with *ninaE*^*G69D*^ to specifically purify *ninaE*^*G69D*^-expressing cell nuclei for cell type-specific gene expression analysis [36]. Sashimi plot visualization of RNA-seq reads showed that exon 4-skipping junction reads were low (154), whereas exon 4-inclusion junction reads were markedly higher (1894), indicating that exon 4-containing isoforms are predominant in these cells. The relative ratio of exon-exon junction usage appeared comparable between control *GMR-Gal4*/+ and the ER stressed *GMR-Gal4 > UAS-ninaE*^*G69D*^ samples (Fig 1B). We further quantified the composition of *Xrp1*’s splice isoforms by calculating Percent Spliced In (PSI) and Percent Spliced Out (PSO) values for exon 4, similar to other studies that examined *Xrp1* alternative splicing in the context of cell competition [29,30]. PSI represents the fraction of transcripts that include exon 4, whereas PSO is the percentage of transcripts without exon 4. Thus, a high PSI value indicates a higher inclusion of the exon 4 sequence in transcripts, while a lower PSI value indicates a lower ratio of those transcripts. We found no significant difference in PSI between control and ER stress conditions (Fig 1C). The exon 4-including isoforms remained the predominant transcripts in both conditions, with exon 4 inclusion at approximately 85% of the transcripts. These data show that the regulation of Xrp1 activity under ER stress does not involve changes in the level of transcription or splice isoform selection. Xrp1 induction by ER stress is likely mediated by downstream mechanisms such as translational or post-translational control.

### The reporter experiments show that *Xrp1* F and G isoforms contain inhibitory 5’ leaders

**Fig 2:**
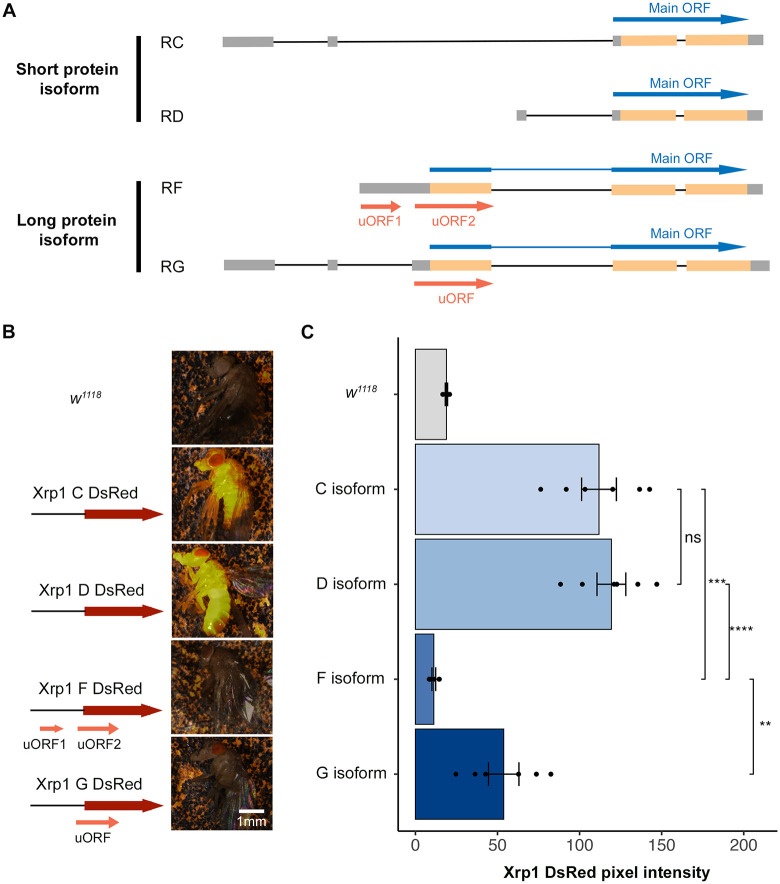
Xrp1 F and G isoforms contain inhibitory 5’ leaders. **(A)** Schematic representation of *Xrp1* transcript isoforms RC, RD, RF, and RG. RC and RD encode the short protein isoform (Xrp1^short^), whereas RF and RG encode the long isoform (Xrp1^long^). The main open reading frame (ORF) is shown in blue. Putative upstream open reading frames (uORFs) present in RF and RG are indicated in orange; uORF2 in RF overlaps the main ORF. **(B)** Representative images of transgenic adult flies expressing the DsRed reporters (red) driven by the *Actin-Gal4* driver with 5′ leader sequences from different Xrp1 isoforms under basal conditions without exogenously imposed stress. To the left of the representative images are the schematic diagrams of the DsRed reporters, where the DsRed ORF is depicted in red and uORFs in orange. Scale bar = 1mm. **(C)** Quantification of DsRed fluorescence intensities of whole adult flies as shown in the representative images of **(B)**. Bars are aligned with the images in (B) and represent mean ± SEM with individual biological replicates overlaid. Expression from uORF-containing isoforms (F and G) is significantly reduced compared to short isoforms (C and D) (one-way ANOVA followed by Tukey’s multiple comparisons test; **p < 0.01; ns, not significant).

We next examined whether any differences in the 5′ leaders of *Xrp1* isoforms could contribute to differential regulation of *Xrp1* expression. We noted that the abundantly expressed long isoforms that transcribe exon 4 of *Xrp1* contain AUG codons upstream of the main ORF in the 5’ leader, possibly encoding upstream open reading frames (uORFs) [36]. The short *Xrp1* isoforms lack these features, with the first AUG codon at the main ORF (Fig 2A). In particular, the RF isoform has two putative uORFs, with the second uORF (uORF2) overlapping with the main ORF. The RG isoform has one uORF which also overlaps with the main ORF.

A vast number of translated uORFs have been identified in cellular mRNAs but only some affect the translation of the main ORF [15]. We tested if the uORF-containing 5’ leaders of Xrp1 affect the expression of the main ORF under basal conditions without exogenously imposed stress. Specifically, we generated DsRed reporter constructs in which the main coding sequence of individual Xrp1 isoforms (C, D, F, and G) was replaced with DsRed (Figs 1S and 2B). These reporters were expressed through the *Actin-Gal4/UAS* system to measure the DsRed expression in the whole fly bodies. Reporters corresponding to the short isoforms that lack uORFs (C and D) exhibited robust DsRed expression. In contrast, reporters derived from the long isoforms (F and G), which contain uORFs, showed markedly reduced or undetectable DsRed signals. Quantification of DsRed intensity confirmed a significant decrease in expression from uORF-containing isoforms (Fig 2C). Together, these data indicate that Xrp1 uORF-containing 5’ leaders can inhibit main ORF expression.

### The expression of HA reporters indicates that the uORF2 of Xrp1’s F isoform inhibits the main ORF translation

**Fig 3:**
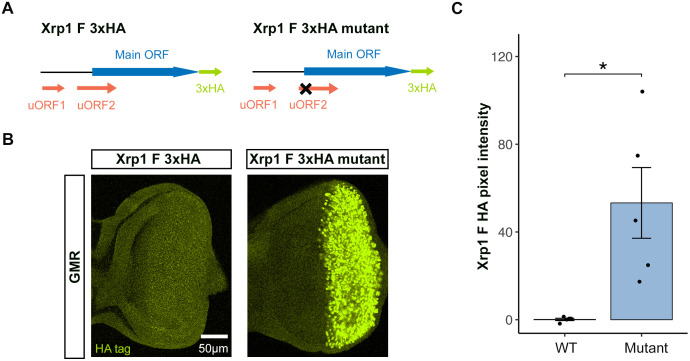
The HA reporter transgenes indicate that the uORF2 of Xrp1 F inhibits the main ORF expression. **(A)** Schematic of Xrp1 F 3xHA constructs. The Xrp1 F isoform contains two putative uORFs (uORF1 and uORF2) upstream of the main ORF. 3xHA epitope tags were placed C-terminal to the Xrp1 coding sequence. In the mutant construct, the start codon of uORF2 is mutated to disrupt its translation without affecting the main ORF. **(B)** Representative confocal images of eye imaginal discs expressing Xrp1 F 3xHA or the uORF2 mutant under basal conditions without experimentally imposed stress, stained for HA (green). Anterior is to the left and posterior to the right. The wild-type Xrp1 construct shows minimal HA signal, whereas the uORF2 mutant exhibits strong HA expression in the *GMR-Gal4*-active regions of the posterior eye discs. Scale bar = 50 µm. **(C)** Quantification of HA fluorescence intensity. Bars represent mean ± SEM with individual biological replicates overlaid. Mutation of uORF2’s AUG codon significantly increases Xrp1 F expression (two-tailed Student’s t-test; *p < 0.05).

Since the two inhibitory 5’ leaders of Xrp1 correlate with the presence of uORFs, we specifically tested if uORFs inhibit main ORF expression. We focused on uORF2 as it overlaps with the main ORF in a different reading frame. During scanning from the 5’ cap along the *Xrp1* mRNA, ribosomes will encounter the uORF2 AUG prior to reaching the main ORF, translating uORF2 at the expense of the main ORF. If this is true, mutating the AUG of uORF2 would relieve this inhibition and enhance translation of Xrp1 main ORF. To test this, we generated an Xrp1 reporter construct in which Xrp1 F isoform 5’ leader was placed before the full-length Xrp1^long^ coding sequence tagged with 3xHA at the C terminus (Fig 3A). We compared the expression of this Xrp1 wild-type reporter with a mutant construct that has the ATG start codon of uORF2 substituted to CCG in an otherwise identical background. Immunostaining for the HA tag revealed little to no detectable signal in tissues expressing the wild-type Xrp1 F construct, indicating minimal translation of the main ORF under basal conditions (Fig 3B). In contrast, mutation of uORF2 resulted in a strong increase in HA signal, demonstrating robust expression of Xrp1 F. Quantification of HA fluorescence intensity indicated a significant difference in expression for the two constructs (Fig 3C). These data support the idea that ribosomes scan along the Xrp1 5’ leader under normal conditions, and uORF2’s AUG codon interferes with the translation of the main ORF.

### Xrp1 induction by *ninaE*^*G69D*^ expression in eye discs is regulated by both the 5’UTR and Xrp1’s main coding sequence

**Fig 4:**
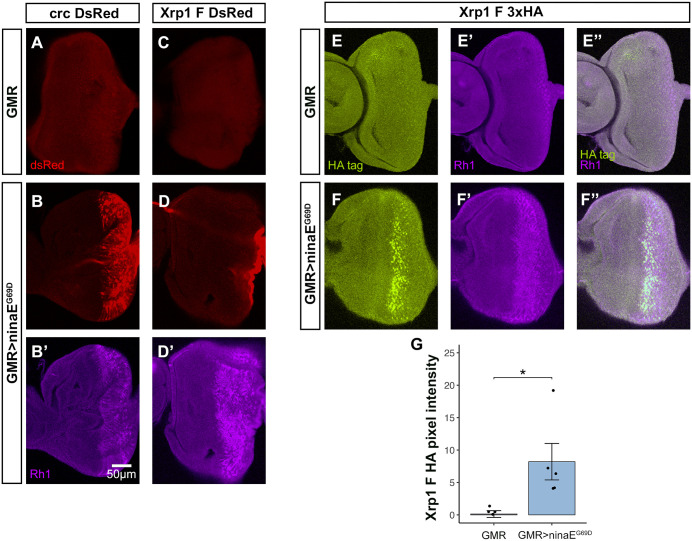
Stress-induced Xrp1 expression involves both the 5′ UTR and coding sequence. **(A-D)** Representative images of eye imaginal discs expressing DsRed reporters under the *GMR-Gal4* driver. Anterior is to the left and posterior is to the right in all panels. DsRed is shown in red. **(A, C)** Eye discs under basal conditions without experimentally imposed stress. **(B, D)** GMR>*ninaE*^*G69D*^ eye discs that express the ER stress-imposing *ninaE*^*G69D*^ (encoding a mutant Rh1) in the posterior eye discs. **(B’, D’)** Anti-Rh1 single channel (magenta) of eye discs shown in **(B, D)**. **(C, D)** The Xrp1 F DsRed reporter retains the endogenous 5′ leader but lacks the Xrp1 coding sequence. The DsRed signal remains low in both the control disc without stress **(C)** and those with ER stress **(D)**. The red fluorescence at the posterior tip of the eye disc is a background signal coming from the M{3xP3-RFPattP} element associated with the *uas-ninaE*^*G69D*^ chromosome. In contrast to Xrp1 F DsRed, the crc DsRed reporter shows a clear induction in the entire domain of *ninaE*^*G69D*^-expressing cells **(A, B)**. **(E, F)** Representative confocal images of eye imaginal discs expressing Xrp1 F 3xHA (green) under control **(E)** and ER stress (*GMR>ninaE*^*G69D*^) conditions **(F)**. **(E’, F’)** Anti-Rh1 single channel. **(E,” F”)** Merged channel images. Anti-HA signal is low under basal conditions and notably induced under ER stress. Scale bar = 50µm. **(G)** Quantification of HA fluorescence intensity. Bars represent mean ± SEM with individual biological replicates overlaid. Xrp1 F 3xHA expression is significantly induced in discs with ER stress imposed by *GMR>ninaE*^*G69D*^ relative to the negative control not expressing *ninaE*^*G69D*^ (two-tailed Student’s t-test; *p < 0.05).

In yeast GCN4 and metazoan ATF4, uORFs allow preferential translation of the main ORFs in response to stress while inhibiting their translation in unstressed cells [14–19]. This regulatory potential of ATF4 5’ leader can be visualized in *Drosophila* with a reporter in which the 5’ leader of the *Drosophila* ATF4 (crc) is placed upstream of the DsRed ORF [5]. This DsRed reporter is induced when ER stress is imposed in the posterior eye discs through the expression of *ninaE*^*G69D*^ (*GMR>ninaE*^*G69D*^) (Fig 4A and 4B). To determine whether Xrp1 5′ leader sequence has similar properties, we examined an equivalent DsRed reporter with the 5’ leader of the Xrp1 F isoform instead of the *crc* 5’ leader. The DsRed expression of this Xrp1 reporter was minimal, both under basal conditions and in *ninaE*^*G69D*^-expressing eye imaginal discs (Fig 4C and 4D). These results indicate that, unlike ATF4 (crc), the 5′ leader of Xrp1 F isoform alone is not sufficient to drive stress-induced expression.

Since the 5’ leader of Xrp1 is insufficient to mediate Xrp1 induction under stress, we asked whether the coding sequence of Xrp1 also contributes to its induction by examining the transgene containing the full-length Xrp1 F 5’ leader and the coding sequence fused to a C-terminal 3xHA tag. This HA-tagged Xrp1 reporter had minimal expression under basal conditions without stress, consistent with the inhibitory effect of the Xrp1 5’ leader (Fig 4E). But in contrast to the Xrp1 5’ leader-DsRed reporter, the HA-tagged Xrp1 F reporter that includes the main ORF showed a reproducible induction in the anterior part of the experimental domain where stress was imposed with *ninaE*^*G69D*^-expression (Fig 4F and 4G). The equivalent HA-tagged Xrp1 F transgene with the uORF2 AUG mutated was strongly expressed both under basal conditions (Fig 3B) and under conditions of stress caused by *GMR>ninaE*^*G69D*^ (S2 Fig). These results show that, although Xrp1 F isoform is regulated by a uORF-based mechanism similar to the canonical stress-responsive transcription factors ATF4 and GCN4, Xrp1 induction is additionally regulated by its own main ORF sequence.

### The pseudopupil analysis and semithin sections show that *Xrp1* is required to prevent light-induced retinal degeneration

**Fig 5:**
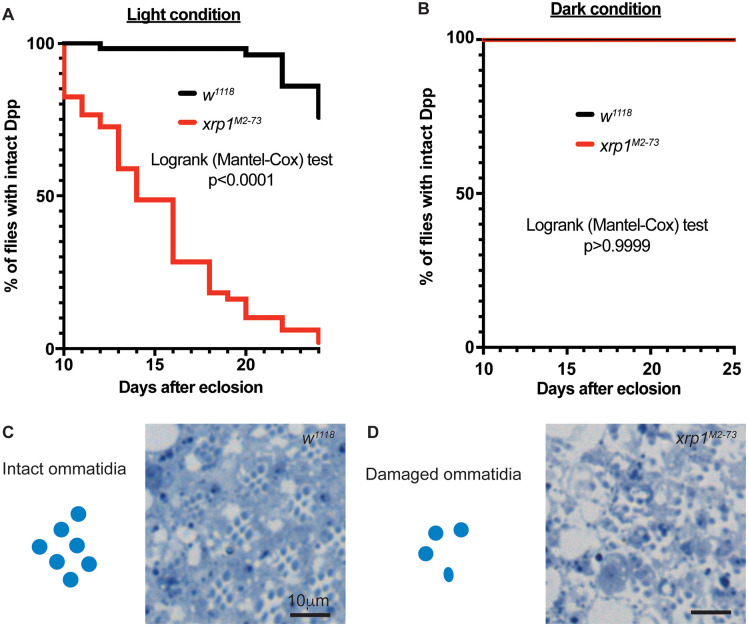
The pseudopupil and semithin sections show that *Xrp1* is required to prevent light-induced retinal degeneration. **(A)** Retinal integrity as assessed by deep pseudopupils (Dpp) in flies exposed to constant light exposure (240-258 Lux white light) for 24 days. *w*^*1118*^ (control, n = 56) and *w*^*1118*^*; Xrp1*^*M2-73*^ (n = 51) were compared. Dpp was specifically examined starting day 10 until day 24 post-eclosion. Two-sided Log-rank (Mantel-Cox) test was used to calculate the p-value. **(B)** An equivalent Dpp assessment in flies reared in dark. **(C, D)** Semithin section images of retina from Day 20 post-eclosion adult flies stained with toluidine. Left of the images are schematic diagrams of rhabdomere patterns in an ommatidia. **(C)** The retina of *w*^*1118*^ (control). **(D)** *w*^*1118*^*; Xrp1*^*M2-73*^ mutant flies. Scale bar = 10µm.

To examine the role of *Xrp1* in a more physiologically relevant setting, we turned to adult *Drosophila* photoreceptors that express endogenous *ninaE* transcripts. Light exposure is a source of stress for these photoreceptors and their integrity is dependent on ER quality control factors and ISR mediators including PERK and ATF4 (crc) [4,6]. To test if *Xrp1* also affects photoreceptor structures, we examined *Xrp1*^*M2-73*^ mutant flies [38], which has a premature stop codon early in the coding sequence. We first assessed the integrity of the *Drosophila* retina by examining deep pseudopupils (Dpp), a trapezoidal image that forms deep in the retina by the projection of regular photoreceptor arrays from many ommatidial clusters [39] (Fig 5). We specifically found that most *Xrp1* mutant flies reared under constant light exposure (240-258 Lux) lost Dpps by 20 days after eclosion, while more than 90% of control flies (*w*^*1118*^) reared under equivalent conditions had visible Dpps (Fig 5A). The phenotype was light-dependent, as *Xrp1* mutant flies reared in the dark did not show the loss of Dpps (Fig 5B). The Dpp loss phenotype in flies reared under light was further validated through the semithin section images of adult fly eyes, which revealed disorganized ommatidial structures specifically in the mutant flies at Day 20 (Fig 5C and 5D). These results indicate that *Xrp1* has a protective effect on the *Drosophila* eye exposed to chronic light stress.

### The reporter experiments suggest that *parkin* regulates Xrp1 expression and *Xrp1* loss reduces the lethality of *parkin* mutants after eclosion

**Fig 6:**
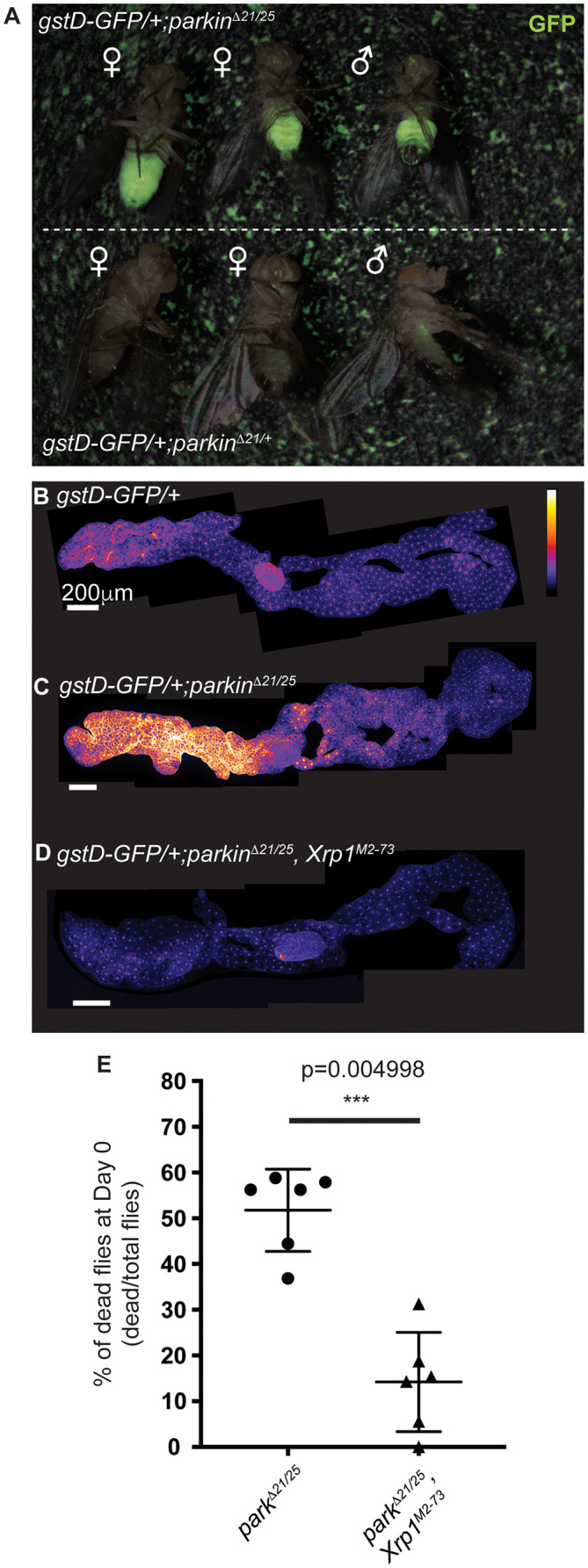
Xrp1 regulation and function in *parkin* mutant flies. **(A)** Adult flies of the indicated genotypes with one copy of the gstD-GFP reporter (green). The top three flies are *parkin*^*Δ21*^*/park*^*25*^ trans-heterozygotes (two females on the left and one male on the right). The bottom three flies are *parkin*^*Δ21/+*^ , which serve as controls. Note strong gstD-GFP induction in the abdomen of the *parkin* trans-heterozygous flies. **(B-D)** The expression of the Xrp1 reporter, gstD-GFP, in the male fat bodies of the wandering third instar **(B)** control *gstD-GFP/ +* . **(C)** *parkin*^*Δ21*^*/parkin*^*25*^ mutant. **(D)** *parkin*^*Δ21*^, *Xrp1*^*M2-73*^*/parkin*^*25*^, *Xrp1*^*M2-73*^ double mutant. The signal level is visualized by the heat map. Warmer color indicates high expression, and cold color is low expression. Left is posterior, and right is anterior. **(E)** The death rate of Day 0 adult fly in *parkin*^*Δ21*^*/parkin*^*25*^ (n = 105) and *parkin*^*Δ21*^, *Xrp1*^*M2-73*^*/parkin*^*25*^, *Xrp1*^*M2-73*^ double mutants (n = 87). The rate is calculated as a ratio of dead flies/total flies*100. P-value was calculated using Wilcoxon rank sum test.

*Parkin* loss is associated with an early onset Parkinson’s disease and the *Drosophila parkin* mutants show excessive ATF4 signaling in many tissues, including the fat body [5,13,40]. *Parkin* encodes an E3 ubiquitin ligase, and together with PINK, helps to remove damaged mitochondria through mitophagy [12]. To test if Xrp1 is also active in *Drosophila parkin* mutants, we examined gstD-GFP, an Xrp1 target reporter [36]. We found a dramatic induction of the gstD-GFP reporter expression in *parkin* mutant adult abdomen (Fig 6A). In the developing third instar larvae, gstD-GFP was expressed broadly at basal levels in the fat body of control *Drosophila* larvae, with slightly higher GFP levels seen in the posterior region. In the *parkin* homozygous mutant larvae, we found a clear induction of the gstD-GFP reporter expression (Fig 6B and 6C). Such gstD-GFP induction was due to Xrp1, as the reporter levels were suppressed in the *parkin-Xrp1* double mutant larvae (Fig 6D). These data indicate that *parkin* loss leads to the induction of Xrp1-mediated gene expression.

To assess the physiological role of *Xrp1* in *parkin* mutants, we examined the lethality of *parkin* homozygous flies. As reported previously, there was a high mortality rate of *parkin* mutants within a day of eclosion, with almost half of them dead at this time point (Fig 6E). Such mortality was suppressed in the *Xrp1-parkin* double mutant, indicating that Xrp1 contributes to the *parkin* mutant phenotype.

## Discussion

Xrp1 has drawn significant interest in recent years due to its association with cell competition and several degenerative disease models [22,23,29–35,41–43]. Xrp1’s activation mechanism has been more extensively studied in *Drosophila* models of cell competition, where recent findings indicate that *Xrp1* undergoes alternative splicing in Rp heterozygous cells, generating Xrp1 isoforms that lack inhibitory 5’ UTRs [29]. Here, we report that ER stress in the eye imaginal discs induces Xrp1 through a distinct mechanism. In this context, there is no significant change in the Xrp1 splice isoform composition, and the uORF-containing isoforms remain predominant. Instead, the second uORF inhibits the main ORF expression in unstressed cells, and Xrp1 induction during ER stress is regulated by both the uORF-containing 5’ leader and the main ORF sequence.

Previous studies had noted a few differences in the regulation of Xrp1 between models of cell competition and ER stress. In Rp mutation-induced cell competition, Xrp1 functions genetically upstream of eIF2α phosphorylation [33–35]. On the other hand, in eye imaginal discs under ER stress, Xrp1 is induced downstream of the eIF2α kinase, PERK [36]. In addition, Rp mutation-induced cell competition requires RpS12 to activate Xrp1 [22,27,28], and recent studies suggest that RpS12 promotes Xrp1 activation through mRNA alternative splicing, increasing the amount of Xrp1 isoforms that lack uORFs [29,30]. In contrast, the ER stress response mechanism associated with phospho-eIF2α and the uORF-mediated ATF4 induction has been extensively characterized and does not currently implicate RpS12. Specifically, it is established that phospho-eIF2α binds and inhibits the eIF2B complex, which normally acts as a GEF to assemble the eIF2-GTP-Met-tRNA_i_ ternary complex (TC). This reduces the TC availability and strongly impacts mRNAs with uORFs, as the scanning ribosomes need to re-acquire another TC to reinitiate translation after uORF translation. Delayed TC reloading can cause some scanning ribosomes to skip the AUG of uORF2, thus allowing translation of downstream main ORF [15,44]. In this widely accepted translational control mechanism, there is little room for a regulatory alternative splicing event. Based on these observations, we propose that Xrp1 is regulated by fundamentally distinct mechanisms depending on the stress context. In cell competition, Xrp1 induction appears to rely on RpS12-dependent alternative splicing, whereas in ER stress, it is regulated by uORF-mediated translational control.

The current study further characterizes the regulatory features of the Xrp1 F isoform 5’ leader. The position of the two uORFs is similar to that found in ATF4, with the second uORF overlapping with the main ORF in a different reading frame [18,19,45]. Consistent with ATF4, we find that the second uORF represses translation of the main ORF in unstressed cells. What was unexpected is the involvement of the Xrp1 main ORF sequence during the ER stress response. A DsRed reporter lacking the main ORF sequence failed to be induced by ER stress in eye discs, whereas an HA-tagged construct containing the full coding sequence was clearly induced. It is unclear how the main ORF impacts Xrp1’s inducibility. As Xrp1 is an auto-activating transcription factor, the protein encoded by the main ORF can further induce the expression of the endogenous Xrp1 allele. However, our experimental design using the Gal4/UAS-based assay to assess the HA-epitope of the transgenic Xrp1 would not be affected by this autoregulatory loop. It is possible that the main ORF sequence contributes to mRNA secondary structure, thereby affecting its expression. Recent studies have shown that mammalian ATF4 mRNA forms a stem loop structure with the main ORF sequence, which is involved in the fine tuning of its inducibility [46]. However, we were unable to identify clear regulatory elements using the RNA secondary structure prediction tools. The precise mechanism associated with Xrp1 F isoform induction remains to be investigated.

In addition to its regulatory mechanism, we identify previously unrecognized roles of Xrp1 in two distinct disease models. First, loss of *Xrp1* results in age-dependent photoreceptor degeneration, even in the absence of additional genetic stress. This is consistent with our previous observation that many *Drosophila* cell types experiencing physiological stress require the activity of stress response pathways [47]. The adult *Drosophila* photoreceptors are particularly sensitive to the loss of ISR mediators such as PERK and crc (ATF4), supporting the idea that cells rely on stress response pathways for homeostasis [4,5]. Our findings suggest that *Xrp1* similarly contributes to the maintenance of photoreceptor homeostasis under physiological stress.

We have also examined *Drosophila parkin* mutants, which experience mitochondrial stress and activate ISR signaling. While previous studies focused on the roles of PERK and crc (ATF4) in this model [5,13], we show that Xrp1 activity, assessed using a gstD-GFP reporter, is elevated in multiple tissues, including the larval fat body. Whereas *Xrp1* plays a protective role in adult photoreceptors, loss of *Xrp1* improves viability in *parkin* mutants during adult eclosion. Thus, the role of *Xrp1* appears to be context dependent.

In summary, we report a distinct Xrp1 regulatory mechanism in eye imaginal disc cells under ER stress and demonstrate its role in two degenerative disease models. The results indicate that Xrp1 induction is governed by different regulatory mechanisms depending on the source of stress and that Xrp1 can have both protective and aggravating roles in different disease models. These results provide new insights into the regulation and physiological functions of Xrp1.

## Materials and methods

### Fly genetics

All flies were reared in standard cornmeal agar medium and maintained at room temperature (20–23 °C). For gene expression experiments, the following lines were used: *Xrp1*^*M2-7*^ [38], *parkin*^*Δ21*^, *parkin*^*25*^ [48,49], *Actin 5C-Gal4/CyO* (Bloomington Stock Center #4414), *GMR-Gal4* [50]*, uas-ninaE*^*G69D*^ [7], *uas-KASH-GFP* [51].

Xrp1 F DsRed reporter constructs were generated by replacing the Xrp1 coding sequence with DsRed while retaining the endogenous 5′ leader sequences. The resulting constructs were subcloned into the pUAST-attB plasmid, which provides a SV40 3’ UTR for all transcripts. The plasmids were injected into PBac{y[+]-attP-3B}VK00037 (Bloomington Drosophila Stock Center #9952) for phiC31-mediated integration into the second chromosome (injected by BestGene Inc.).

Xrp1 F 3xHA constructs were made with full length Xrp1 coding sequences fused in frame to a C-terminal 3xHA tag. The Xrp1 F 3xHA mutant construct was made by changing the start codon of uORF2 from ATG to CCG. These constructs were subcloned into the pUAST-attB plasmid, which provides a SV40 3’ UTR. The plasmids were injected into PBac{y[+]-attP-3B}VK00037 (Bloomington Drosophila Stock Center #9952) for phiC31-mediated integration into the second chromosome (BestGene Inc.).

### Alternative splicing analysis

Alternative splicing analysis was performed with the previously published sequencing data [36] with the Alternative Splicing and Transcript Tools (VAST-TOOLS) v2.2.2 (https://github.com/vastgroup/vast-tools) following the approach described in [30]. RNA-seq reads were aligned and processed against the *Drosophila melanogaster* reference genome (dm6). Splicing events were quantified using the VAST-TOOLS pipeline, which calculates Percent Spliced In (PSI) values for exon inclusion. Percent Spliced Out (PSO) values were calculated as 100 − PSI. Statistical significance was assessed using two-tailed *t*-tests unless otherwise stated. Graphs were generated using the ggplot2 package in the R programming environment. Sashimi plots were generated using the Integrative Genomics Viewer (IGV) from aligned BAM files (dm6 genome). These plots display exon coverage and exon-exon junction reads, with values representing the total number of reads across three biological replicates per condition. A minimum junction coverage threshold of 30 reads was applied.

### DsRed fluorescence imaging in whole fly bodies

Flies were generated by crossing UAS-DsRed lines to *Actin-Gal4* drivers. Male F1 progeny (0–3 days post-eclosion) were collected for analysis. Whole flies were imaged using a Nikon SMZ1500 stereomicroscope. DsRed fluorescence images were acquired using identical settings across all samples, including a fixed exposure time of 5 s. Corresponding brightfield images were captured under transmitted light using standard acquisition settings.

### Fluorescence microscopy, immunohistochemistry, and antibodies

Whole flies expressing fluorescent reporters of Xrp1 and gstD-GFP (Figs 2D, S1 and S2) were imaged with a Nikon SMZ1500 microscope and the associated NIS Element software while the flies were on the CO_2_ gas pad.

We followed standard protocols for whole-mount immunofluorescence. Imaginal discs were fixed in 1 X PBS containing 0.2% Triton X-100 (Millipore-Sigma, cat #T8787) (PBT x 0.2%) and 4% paraformaldehyde (Alfa Aesar, cat #43368) for 25 min at ambient temperature with gentle rocking. Following fixation, discs were rinsed three times, 3 x 10 mins, in PBT x 0.2% and then incubated with primary antibodies diluted in PBT x 0.2% for 1 hour at ambient temperature with gentle rocking. After primary antibody incubation, discs were washed 3 x 10 mins with PBT x 0.2% and incubated with secondary antibodies diluted in PBT x 0.2% for 1 hour at ambient temperature with gentle rocking, covered from light. For larval fat body immunofluorescence, rabbit anti-GFP (Invitrogen cat #6455 1:500) was used prior to incubation with a secondary antibody. Samples were then washed 3 x 10 mins in PBT x 0.2%, covered from light, then mounted in VECTASHIELD HardSet Antifade Mounting Medium (Vector Laboratories, cat #H1400). Confocal images were acquired using a Zeiss LSM 700 confocal microscope (Carl Zeiss) with a 20X objective.

For DsRed fluorescence imaging, primary antibodies used are mouse anti-Rh1 (1:500, Developmental Studies Hybridoma Bank (DSHB), 4C5 concentrate) and rabbit anti-DsRed (1:1000, Takara Bio cat #632496). Secondary antibodies used are Alexa Fluor 546 goat anti-mouse (1:500, Invitrogen, cat #A11003), and Alexa Fluor 488 goat anti-rabbit (1:500, Invitrogen, cat #A32371).

For HA fluorescence imaging, primary antibodies used are mouse anti-Rh1 (1:500, Developmental Studies Hybridoma Bank (DSHB), 4C5 concentrate), rabbit anti-HA (1:100, Cell Signaling cat #3724), and guinea pig anti-Xrp1 (1:1000, previously generated in our lab). Secondary antibodies used are Alexa Fluor 546 goat anti-mouse (1:500, Invitrogen, cat #A11003), Alexa Fluor 488 goat anti-rabbit (1:500, Invitrogen, cat #A32371), and Alexa Fluor 647 goat anti-guinea pig (1:500, Invitrogen, cat #A21450).

### Semithin section images

20 days after eclosion flies were collected to prepare semithin sectioning samples. The flies were prepared through the protocol of the microscope core facility in New York University. The semithin sectioning samples were imaged using Zeiss Axio Observer 7 (Carl Zeiss).

### Dpp degeneration assay

Adult flies at 0–1 day after eclosion (AE) were collected (Light; *w*^*1118*^, n = 57, *Xrp1*^M2-73^, n = 51) and reared in regular cornmeal medium at a 25 ˚C throughout the degeneration assay period. Vials were covered with parafilm containing holes to allow gas exchange. Illumination was provided using an LED light pad (B4 Tracing Light Box with Internal Cord + Foldable Stand, 14.2 * 10.6 inches Light Board for Tracing, 3-Levels Brightness, 8000 LUX Tracing Light Pad for Children, VKTEKLAB), positioned on top of a cardboard box during the assay (Width:14cm × Depth:11.5cm × Height:15cm). Light intensity was measured using a Fisherbrand Traceable Dual-Range Light Meter (06-662-63. Fisher scientific) and adjusted to be 240–258 Lux at the bottom of the box. The Dpp phenotype was examined using an SMZ1500 stereomicroscope (Nikon). Dpp was examined under blue-light illumination, which enhanced the visualization of the Dpp structures. Statistical analyses were performed using GraphPad Prism 10 (GraphPad Software).

### Adult death rate analysis

Parkin homozygous mutants exhibited high death rate immediately after adult eclosion, and this was quantified in Fig 6E. Each vial contained flies developed from 20 larvae. The number of dead flies after eclosion were divided by the total number of elosed flies in each vial to calculate the death rate for each vial.

### Quantification of DsRed and HA pixel intensity in the eye imaginal discs

Image analysis was performed using Fiji (https://imagej.net/software/fiji/). Average pixel intensities of DsRed were quantified from whole fly bodies, while HA signal intensities were measured in eye imaginal discs. To present anti-HA pixel intensities in graphs, the anti-HA pixel intensities from the region without *ninaE*^*G69D*^ expression (negative control region) was subtracted from the pixel intensities within the *ninaE*^*G69D*^ expressing domain (ER stressed samples). Fold changes for both DsRed and HA signals were calculated by normalizing average pixel intensities to those of control samples. Statistical significance was assessed using two-tailed t-tests or ANOVA followed by Tukey’s post-hoc test. Graphs were generated using the ggplot2 package in the R programming environment.

## Supporting information

S1 Fig Xrp1 DsRed reporters reveal differential expression across isoforms. **(A-D)** Representative images of adult flies expressing DsRed reporters driven by different Xrp1 isoform 5′ leaders under identical conditions. Schematics above each panel indicate the corresponding Xrp1 isoform and the presence or absence of upstream open reading frames (uORFs). Scale bar = 1mm.
(TIF)

S2 Fig Representative confocal images of eye imaginal discs expressing Xrp1 F 3xHA mutant under ER stress (*GMR>ninaE*^*G69D*^). Anti-HA signal is robustly induced. Scale bar = 50µm.
(TIF)

S1 Data Source Data. Measurement values used to generate graphs and all raw image files used in the figures of the manuscript.
(XLSX)
