# Supplementary figures and images for: The regulation of Xrp1 expression by uORFs and main ORF sequences and its function in Drosophila disease models
Source: PLoS Genet. 2026 Jun 10;22(6):e1012203. doi: 10.1371/journal.pgen.1012203 (PMC13268129; doi:10.1371/journal.pgen.1012203)

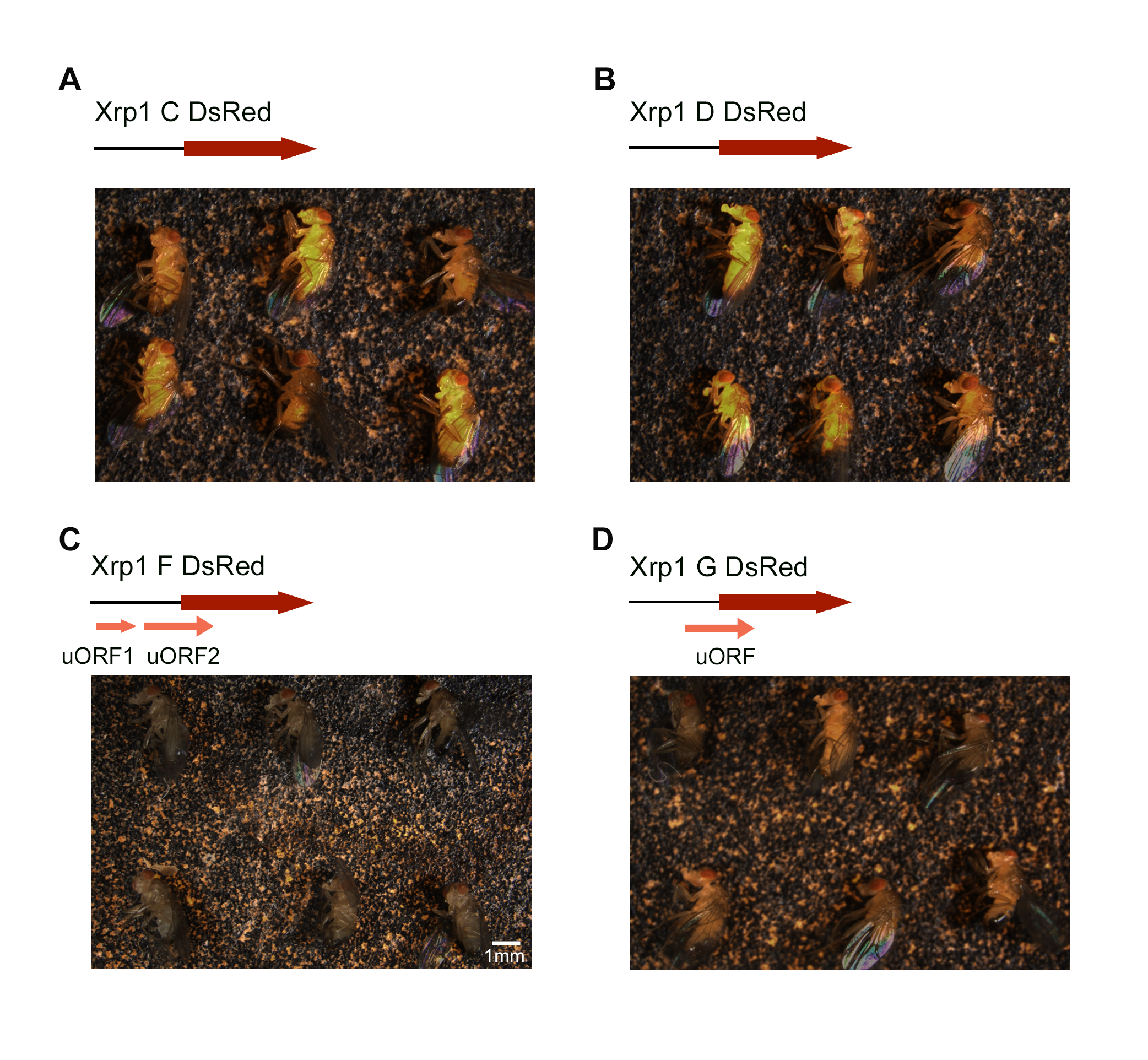

Supplement: S1 Fig — (A-D) Representative images of adult flies expressing DsRed reporters driven by different Xrp1 isoform 5′ leaders under identical conditions. Schematics above each panel indicate the corresponding Xrp1 isoform and the presence or absence of upstream open reading frames (uORFs). Scale bar = 1mm. (TIF) [file pgen.1012203.s001.tif]

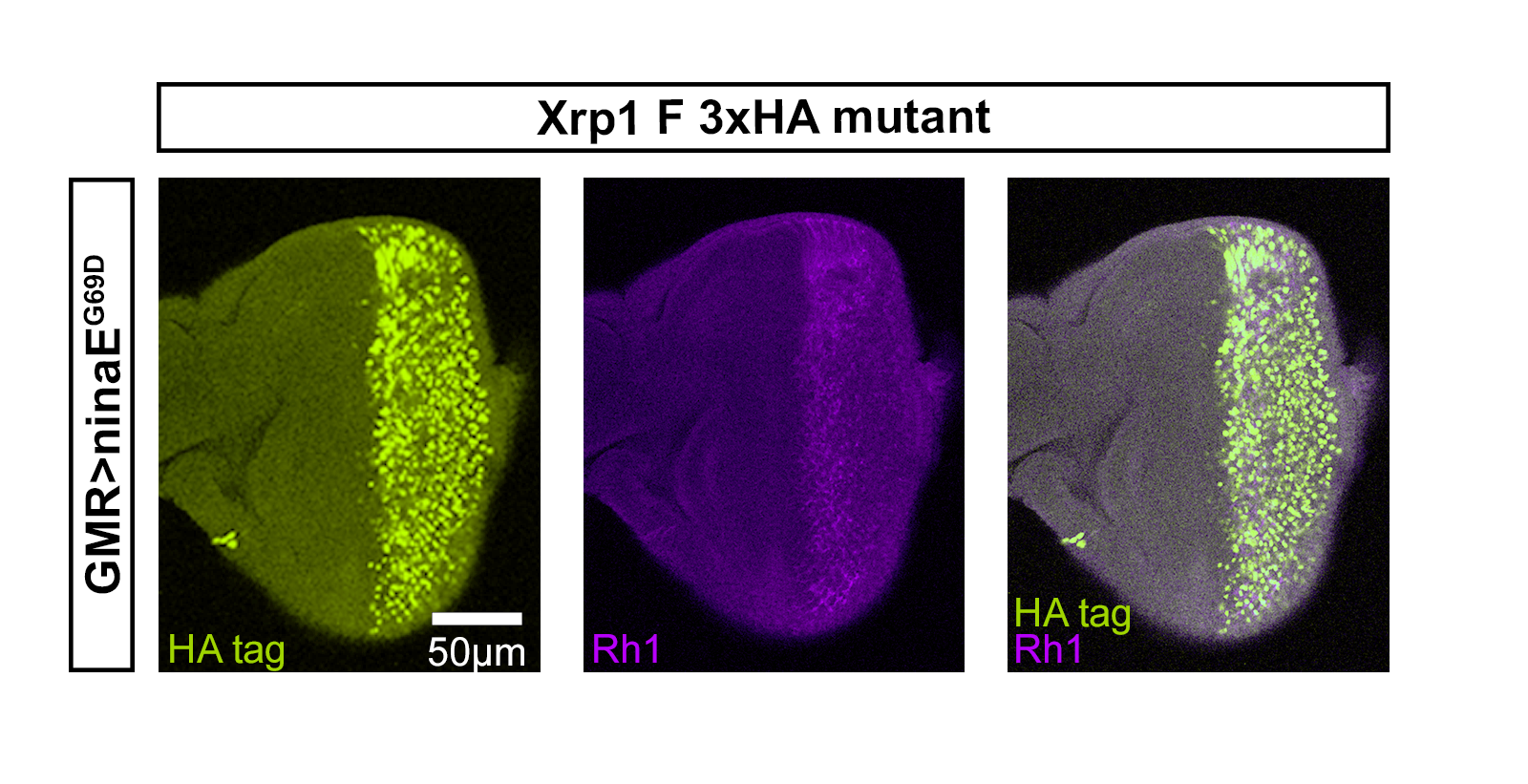

Supplement: S2 Fig — Anti-HA signal is robustly induced. Scale bar = 50µm. (TIF) [file pgen.1012203.s002.tif]
