# Supplementary material for: The regulation of Xrp1 expression by uORFs and main ORF sequences and its function in Drosophila disease models
Source: PLoS Genet. 2026 Jun 10;22(6):e1012203. doi: 10.1371/journal.pgen.1012203 (PMC13268129; doi:10.1371/journal.pgen.1012203)
Supplement: Attachment — Submitted filename: PlosGenetics_response_to_reviewers.pdf [file pgen.1012203.s005.pdf]

Response to Reviewers: PGENETICS-D-26-00363

We thank the reviewers for the positive assessment and suggestions for further improvement. We have revised the manuscript accordingly and below is a summary of the changes. The original reviewer comments are in black, and our responses are marked in blue.

Reviewer #1: This is an interesting manuscript where the authors dissect the function of the uORFs found in the Drosophila gene Xrp1. This gene functions in the ISR, so it's regulation is of interest broadly. The authors discover that a uORF that overlaps the coding sequence indeed functions to inhibit expression. They also show that the function of the Xrp1 gene depends on the nature of the stress. For example, in normal eye development, the Xrp1 gene is protective. But in the parkin mutant, the Xrp1 gene promotes organismal lethality.

Overall this is a nice paper. However, a confusing issue is that sometimes they are looking at basal expression with their constructs, and sometimes they are looking in a misfolded protein context, and of course regulation might be different. it would be helpful to see the expression of all of the constructs quantitated in the eye disc under basal conditions and with the rhodopsin mutant, to compare basal to misfolded protein, at least for the eye disc. In addition, they should clearly indicate those conditions that are basal (Fig 2B,C; figure 3B; parts of figure 4).

Thank you for the point. We agree that the distinction between basal and ER stress conditions was not sufficiently clear in the original submission. We have revised the text accordingly:

- Figure 2B legend is now, “Representative images of transgenic adult flies expressing the DsRed reporters (red) driven by the *Actin-Gal4* driver with 5' leader sequences from different Xrp1 isoforms under BASAL conditions without exogenously imposed stress.”
- Figure 3B legend is now, “Representative confocal images of eye imaginal discs expressing Xrp1 F 3xHA or the uORF2 mutant under BASAL conditions without experimentally imposed stress, stained for HA (green).”
- Figure 4G (previously Figure 4C) legend is now, “Xrp1 F 3xHA expression is significantly induced in discs with ER stress imposed by *GMR>ninaE*^{G69D} relative to the negative control not expressing *ninaE*^{G69D}.”

Similar changes were made in the main text describing the results.

Please note that all Xrp1 reporter levels have been quantified (Figures 2C, 3C, 4G).

lines 234-236 must be incorrect? if not the data are not visible.

The description in the lines 234-236 was referring to the old Figure 4A middle left panel, but the confusion could have been caused by having so many panels under the umbrella of Figure 4A. To avoid confusion, we have now given individual alphabetic labels to all panels in the revised manuscript.

Why is figure S2 shown? it is the same as Figure 3B lower panel (only nicer looking).

Fig.3B shows the basal condition without experimentally imposed stress and Fig. S2 is stressed condition. We have clarified these points in the revised Figure legends.

<u>Reviewer #2</u>: XRP1 is a transcription factor with complex roles and regulation in the context of different stress responses. From past works seemingly contradictory models have arisen suggesting that the XRP1 transcription factor may be either regulated by alternative splicing and act upstream of eIF2alpha phosphorylation or, alternatively, its expression level may be regulated downstream of phosphorylated eIF2alpha as part of the integrated stress response (ISR).
The manuscript by Katow et al aims to disentangle some of this confusion with a focus on XRP1 activation in the context of activating the integrated stress response by ER stress. As tool to induce ER stress, the authors relied on the well-established expression of the folding-challenged rhodopsin1-G69D mutation.
With a set of clear, well documented and quantified experiments the authors show that
(i) RNA splicing is not changed by ISR activation.
(ii) small upstream ORFs present only in a subset of splice versions are critical for ISR induced increases in XRP1 expression
(iii) unlike in the case of ATF4, the uORFS are not sufficient to fully explain IRS-indcinduced expression, but the role of the XRP1-encoding ORF is not obvious and not further investigated
(iv) in a somewhat unrelated set of experiments the authors also document different roles of XRP1 function in preventing light-induced retinal degeneration but enhancing premature death in the context of loss of Parkin function.
The experiments are well documented and quantified and convincingly support the authors claims. I have only a few minor suggestions to improve the writing.

Minor suggestions:
line 108: the "Subsequentially..." does not make sense, following the sentence about the XRP1 null allele.

Thank you for the point. We have revised the sentence to "Conversely, overexpression of Xrp1 induces eIF2α phosphorylation, which activates a feed-forward loop to further enhance Xrp1 expression (Baumgartner et al. 2021; Recasens-Alvarez et al. 2021; Langton et al. 2021; Ochi et al. 2021; Kiparaki et al. 2022)."

line 245: To test if Xrp1 also affects PR viability,...
This should be rephrased as the authors show impressive effects of loss of XRP function on photoreceptor and retina structure, but do not actually directly address viability of the

photoreceptors. Assays of photoreceptor cell death would be necessary to support the claim of reduced PR viability.

Thank you for the point. Following the suggestion, we changed the phrase "To test if Xrp1 also affects PR viability" to "To test if Xrp1 also affects PR structures."

line 289 +319 : "the last uORF"..
the meaning of the "last uORF" is not well defined. This should be rephrased.

Following the suggestion, we changed all references of the "last uORF" to "the second uORF ."

lines 320 ff
It may be worth mentioning that one intuitive possibility of the role of the XRP1 ORF in elevating its expression - based on a potential role as auto-activating transcription factor - is already excluded by the authors' experimental design using GAl4/uas-driven expression.

Thank you for the insightful suggestion. We added a line in the Discussion stating that "As Xrp1 is an auto-activating transcription factor, the protein encoded by the main ORF can further induce the expression of the endogenous Xrp1 allele. However, our experimental design using the Gal4/UAS-based assay would not be affected by this autoregulatory loop."

lines 431 ff
"For HA, signal in the posterior region was calculated by subtracting the region where ninaE G69D is expressed from the rest of the posterior region of the disk without ninaEG69D."
This is confusing. It seems that this should be the other way around: For the HA signal, the signal in the posterior regionregion without ninaEG69D should be subtracted from the whole disk.

Thank you for catching the error. We now write that "To present anti-HA pixel intensities in graphs, the pixel intensities from the region without ninaE ^{G69D} expression (negative control region) was subtracted from the pixel intensities within the ninaE ^{G69D} expressing domain (ER stressed regions)."

Reviewer #3: In this paper, the authors study the regulation of Xrp1 and how it relates to the integrated stress response (ISR) in flies. ISR leads to translation of ATF4, which is regulated by uORFs in the 5' end. In flies, Xrp1 is induced by ISR. In this study, the authors show that Xrp1 induction requires both its uORFs and main ORF sequence. ninaE OE leads to stress and does not change the Xrp1 slice form composition. They find that uORF does not mediate ORF induction alone, but requires main ORF sequence. The authors go on to show Xrp1 was required for PR survival in constant light. Moreover, they find that parkin mutation leads to Xrp1 expression and Xrp1 loss enhanced viability of parkin mutants.

This is an excellent paper, requiring only minor text changes before publication. The conclusion in the abstract is somewhat generic, and the paper would benefit from a stronger summary. I provide detailed evaluation of claims and minor suggestions for text/figure edits here:

Main claims and critique

ER stress does not change Xrp1 transcript composition: The analysis of previously acquired RNA-seq data support this point. For figure 1C, I recommend showing the splice isoforms in the same direction/format, not the mirror format as the authors have presented them. Also, it would aid the reader if figure 1C was aligned with figure 1A for easier comparison.

Thank you for the helpful suggestion. Although Figures 1A and 1C were already displayed in the same direction, we revised the figure layout to improve readability. We repositioned Figure 1C to Figure 1B, thereby placing it adjacent to Figure 1A. We also adjusted the exon scaling/proportions in Figure 1A to better match the presentation of the sashimi plot.

Xrp1 exon 4 encodes an inhibitory 5' leader: The reporter experiments show that the F and G isoform 5' uORFs are sufficient to repress expression in figure 2.

Following the suggestion, we changed the heading (page 9) as "The reporter experiments show that Xrp1 F and G isoforms contain inhibitory 5' leaders." Similarly, the Figure 2 legend was changed to "Xrp1 F and G isoforms contain inhibitory 5' leaders."

uORF2 of Xrp1 F inhibitions main ORF translation: The HA reporter constructs show that the uORF2 of Xrp1 F inhibits translation in figure 3.

Following the suggestion, we changed the Figure 3 legend to "The expression of the HA reporters indicate that the uORF2 of Xrp1 F inhibits the main ORF expression." The subtitle of the main text was similarly changed.

The 5' UTR and main coding sequence of Xrp1 are required for induction by ninaE expression: The reporter construct experiments show that the 5'UTR and main coding sequences together lead to expression in figure 4. I would suggest that the authors change this phrasing to a "sufficiency" argument as they are providing the elements, not knocking them out in the endogenous locus. The authors use this framing on page 11, "Since the 5' leader of Xrp1 was insufficient…". Alternatively, the authors could state the conclusion without making a necessity or sufficiency claim.

Thank you for the suggestion. We have replaced the word "requires" in relation to stress-induced Xrp1 regulation with either "involves" or "regulated by" in pages 3, 11, 12, 14. In page 6 and 7, we now make it clear that the Xrp1 "reporter" induction…requires its 5' leader as well as the main ORF sequence.

Loss of Xrp2 leads to age-related retinal degeneration: The Dpp and semithin section images show that Xrp1 is required to prevent light induced degeneration in figure 5.

Thank you for the suggestion. We revised the Figure 5 legend and the main text accordingly.

We also added a new panel, Figure 5B. This is a graph of Dpp loss in flies reared under dark, which serves as a control for Figure 5A. Although the reviewers had not asked for this experiment, we think

this panel makes it clear that the retinal degeneration phenotype of Xrp1 mutants is light-dependent.

The role of Xrp1 in parkin mutants: The reporter experiments suggest that parkin regulates Xrp1 expression and the epistasis experiment argues that Xrp1 is genetically downstream of parkin.

Thank you for the suggestion. We edited the subtitle of the result section (page 13) as follows: "The reporter experiments suggest that parkin regulates Xrp1 expression and Xrp1 loss reduces the lethality of parkin mutations after eclosion."
