# Supplementary material for: The regulation of Xrp1 expression by uORFs and main ORF sequences and its function in Drosophila disease models
Source: PLoS Genet. 2026 Jun 10;22(6):e1012203. doi: 10.1371/journal.pgen.1012203 (PMC13268129; doi:10.1371/journal.pgen.1012203)
Supplement: Peer review history

# Peer review: Decision Letter 0

**DOI:** 10.1371/journal.pgen.1012203.r001

**Authors:** Yanshan Fang, Fengwei Yu

18 May 2026

PGENETICS-D-26-00363

The regulation of Xrp1 expression by uORFs and main ORF sequences and its function in Drosophila disease models

PLOS Genetics

Dear Dr. Ryoo,

Thank you for submitting your manuscript to PLOS Genetics. After careful consideration, we feel that it has merit but does not fully meet PLOS Genetics's publication criteria as it currently stands. Therefore, we invite you to submit a revised version of the manuscript that addresses the points raised during the review process.

Please submit your revised manuscript by August 18th. If you will need more time than this to complete your revisions, please reply to this message or contact the journal office at plosgenetics@plos.org. When you're ready to submit your revision, log on to https://www.editorialmanager.com/pgenetics/ and select the 'Submissions Needing Revision' folder to locate your manuscript file.

Please include the following items when submitting your revised manuscript:

* A letter that responds to each point raised by the editor and reviewer(s). You should upload this letter as a separate file labeled 'Response to Reviewers'. This file does not need to include responses to any formatting updates and technical items listed in the 'Journal Requirements' section below.

* A marked-up copy of your manuscript that highlights changes made to the original version. You should upload this as a separate file labeled 'Revised Manuscript with Track Changes'.

* An unmarked version of your revised paper without tracked changes. You should upload this as a separate file labeled 'Manuscript'.

If you would like to make changes to your financial disclosure, competing interests statement, or data availability statement, please make these updates within the submission form at the time of resubmission. Guidelines for resubmitting your figure files are available below the reviewer comments at the end of this letter.

As the corresponding author, your ORCID iD is verified in the submission system and will appear in the published article. PLOS supports the use of ORCID, and we encourage all coauthors to register for an ORCID iD and use it as well. Please encourage your coauthors to verify their ORCID iD within the submission system before final acceptance, as unverified ORCID iDs will not appear in the published article. Only the individual author can complete the verification step; PLOS staff cannot verify ORCID iDs on behalf of authors.

We look forward to receiving your revised manuscript.

Kind regards,

Yanshan Fang, Ph.D., M.D.

Academic Editor

PLOS Genetics

Fengwei Yu

Section Editor

PLOS Genetics

Aimée Dudley

Editor-in-Chief

PLOS Genetics

Anne Goriely

Editor-in-Chief

PLOS Genetics

**Additional Editor Comments :**

All of the three reviewers are supportive to this work but have raised some questions. In particular, please perform the additional experiments to address Reviewer 1's question regarding expression levels in different fly backgrounds. The remaining comments pertain to clarifying the description of results, improving data presentation, rephrasing the test, strengthening the discussion, and similar revisions. Please revise manuscript according to the reviewers' suggestions.

**Journal Requirements:**

1) We ask that a manuscript source file is provided at Revision. Please upload your manuscript file as a .doc, .docx, .rtf or .tex. If you are providing a .tex file, please upload it under the item type u2018LaTeX Source Fileu2019 and leave your .pdf version as the item type u2018Manuscriptu2019.

2) Please upload all main figures as separate Figure files in .tif or .eps format. For more information about how to convert and format your figure files please see our guidelines:

https://journals.plos.org/plosgenetics/s/figures

3) We have noticed that you have uploaded Supporting Information files, but you have not included a list of legends. Please add a full list of legends for your Supporting Information files after the references list.

4) Some material included in your submission may be copyrighted. According to PLOS’s copyright policy, authors who use figures or other material (e.g., graphics, clipart, maps) from another author or copyright holder must demonstrate or obtain permission to publish this material under the Creative Commons Attribution 4.0 International (CC BY 4.0) License used by PLOS journals. Please closely review the details of PLOS’s copyright requirements here: PLOS Licenses and Copyright. If you need to request permissions from a copyright holder, you may use PLOS's Copyright Content Permission form.

Please respond directly to this email and provide any known details concerning your material's license terms and permissions required for reuse, even if you have not yet obtained copyright permissions or are unsure of your material's copyright compatibility. Once you have responded and addressed all other outstanding technical requirements, you may resubmit your manuscript within Editorial Manager.

Potential Copyright Issues:.

i) Please confirm (a) that you are the photographer of 2B, 6A, and S1, or (b) provide written permission from the photographer to publish the photo(s) under our CC BY 4.0 license.

5) Please revise your current Competing Interest statement to the standard "The authors have declared that no competing interests exist."

Note: If the reviewer comments include a recommendation to cite specific previously published works, please review and evaluate these publications to determine whether they are relevant and should be cited. There is no requirement to cite these works unless the editor has indicated otherwise.

**Reviewers' comments:**

Reviewer's Responses to Questions

Reviewer #1: This is an interesting manuscript where the authors dissect the function of the uORFs found in the Drosophila gene Xrp1. This gene functions in the ISR, so it's regulation is of interest broadly. The authors discover that a uORF that overlaps the coding sequence indeed functions to inhibit expression. They also show that the function of the Xrp1 gene depends on the nature of the stress. For example, in normal eye development, the Xrp1 gene is protective. But in the parkin mutant, the Xrp1 gene promotes organismal lethality.

Overall this is a nice paper. However, a confusing issue is that sometimes they are looking at basal expression with their constructs, and sometimes they are looking in a misfolded protein context, and of course regulation might be different. it would be helpful to see the expression of all of the constructs quantitated in the eye disc under basal conditions and with the rhodopsin mutant, to compare basal to misfolded protein, at least for the eye disc. In addition, they should clearly indicate those conditions that are basal (Fig 2B,C; figure 3B; parts of figure 4).

lines 234-236 must be incorrect? if not the data are not visible.

Why is figure S2 shown? it is the same as Figure 3B lower panel (only nicer looking).

Reviewer #2: XRP1 is a transcription factor with complex roles and regulation in the context of different stress responses. From past works seemingly contradictory models have arisen suggesting that the XRP1 transcription factor may be either regulated by alternative splicing and act upstream of eIF2alpha phosphorylation or, alternatively, its expression level may be regulated downstream of phosphorylated eIF2alpha as part of the integrated stress response (ISR).

The manuscript by Katow et al aims to disentangle some of this confusion with a focus on XRP1 activation in the context of activating the integrated stress response by ER stress. As tool to induce ER stress, the authors relied on the well-established expression of the folding-challenged rhodopsin1-G69D mutation.

With a set of clear, well documented and quantified experiments the authors show that

(i) RNA splicing is not changed by ISR activation.

(ii) small upstream ORFs present only in a subset of splice versions are critical for ISR induced increases in XRP1 expression

(iii) unlike in the case of ATF4, the uORFS are not sufficient to fully explain IRS-indcinduced expression, but the role of the XRP1-encoding ORF is not obvious and not further investigated

(iv) in a somewhat unrelated set of experiments the authors also document different roles of XRP1 function in preventing light-induced retinal degeneration but enhancing premature death in the context of loss of Parkin function.

The experiments are well documented and quantified and convincingly support the authors claims. I have only a few minor suggestions to improve the writing.

Minor suggestions:

line 108: the "Subsequentially..." does not make sense, following the sentence about the XRP1 null allele.

line 245: To test if Xrp1 also affects PR viability,...

This should be rephrased as the authors show impressive effects of loss of XRP function on photoreceptor and retina structure, but do not actually directly address viability of the photoreceptors. Assays of photoreceptor cell death would be necessary to support the claim of reduced PR viability.

line 289 +319 : "the last uORF"..

the meaning of the "last uORF" is not well defined. This should be rephrased.

lines 320 ff

It may be worth mentioning that one intuitive possibility of the role of the XRP1 ORF in elevating its expression - based on a potential role as auto-activating transcription factor - is already excluded by the authors' experimental design using GAl4/uas-driven expression.

lines 431 ff

"For HA, signal in the posterior region was calculated by subtracting the region where ninaE G69D is expressed from the rest of the posterior region of the disk without ninaEG69D."

This is confusing. It seems that this should be the other way around: For the HA signal, the region without ninaEG69D should be subtracted from the whole disk.

Reviewer #3: In this paper, the authors study the regulation of Xrp1 and how it relates to the integrated stress response (ISR) in flies. ISR leads to translation of ATF4, which is regulated by uORFs in the 5’ end. In flies, Xrp1 is induced by ISR. In this study, the authors show that Xrp1 induction requires both its uORFs and main ORF sequence. ninaE OE leads to stress and does not change the Xrp1 slice form composition. They find that uORF does not mediate ORF induction alone, but requires main ORF sequence. The authors go on to show Xrp1 was required for PR survival in constant light. Moreover, they find that parkin mutation leads to Xrp1 expression and Xrp1 loss enhanced viability of parkin mutants.

This is an excellent paper, requiring only minor text changes before publication. The conclusion in the abstract is somewhat generic, and the paper would benefit from a stronger summary. I provide detailed evaluation of claims and minor suggestions for text/figure edits here:

Main claims and critique

ER stress does not change Xrp1 transcript composition: The analysis of previously acquired RNA-seq data support this point. For figure 1C, I recommend showing the splice isoforms in the same direction/format, not the mirror format as the authors have presented them. Also, it would aid the reader if figure 1C was aligned with figure 1A for easier comparison.

Xrp1 exon 4 encodes an inhibitory 5’ leader: The reporter experiments show that the F and G isoform 5’ uORFs are sufficient to repress expression in figure 2.

uORF2 of Xrp1 F inhibitions main ORF translation: The HA reporter constructs show that the uORF2 of Xrp1 F inhibits translation in figure 3.

The 5’ UTR and main coding sequence of Xrp1 are required for induction by ninaE expression: The reporter construct experiments show that the 5’UTR and main coding sequences together lead to expression in figure 4. I would suggest that the authors change this phrasing to a “sufficiency” argument as they are providing the elements, not knocking them out in the endogenous locus. The authors use this framing on page 11, “Since the 5’ leader of Xrp1 was insufficient…”. Alternatively, the authors could state the conclusion without making a necessity or sufficiency claim.

Loss of Xrp2 leads to age-related retinal degeneration: The Dpp and semithin section images show that Xrp1 is required to prevent light induced degeneration in figure 5.

The role of Xrp1 in parkin mutants: The reporter experiments suggest that parkin regulates Xrp1 expression and the epistasis experiment argues that Xrp1 is genetically downstream of parkin.

**********

**Have all data underlying the figures and results presented in the manuscript been provided?**

Large-scale datasets should be made available via a public repository as described in the *PLOS Genetics*    data availability policy, and numerical data that underlies graphs or summary statistics should be provided in spreadsheet form as supporting information.

Reviewer #1: Yes

Reviewer #2: Yes

Reviewer #3: Yes

**********

PLOS authors have the option to publish the peer review history of their article (what does this mean?). If published, this will include your full peer review and any attached files.

If you choose “no”, your identity will remain anonymous but your review may still be made public.

**Do you want your identity to be public for this peer review?**    For information about this choice, including consent withdrawal, please see our Privacy Policy.

Reviewer #1: No

Reviewer #2: No

Reviewer #3: **Yes:**   Robert J Johnston

[NOTE: If reviewer comments were submitted as an attachment file, they will be attached to this email and accessible via the submission site. Please log into your account, locate the manuscript record, and check for the action link "View Attachments". If this link does not appear, there are no attachment files.]

**Figure resubmission:**

While revising your submission, we strongly recommend that you use PLOS’s NAAS tool (https://ngplosjournals.pagemajik.ai/artanalysis) to test your figure files. NAAS can convert your figure files to the TIFF file type and meet basic requirements (such as print size, resolution), or provide you with a report on issues that do not meet our requirements and that NAAS cannot fix.

After uploading your figures to PLOS’s NAAS tool - https://ngplosjournals.pagemajik.ai/artanalysis, NAAS will process the files provided and display the results in the "Uploaded Files" section of the page as the processing is complete. If the uploaded figures meet our requirements (or NAAS is able to fix the files to meet our requirements), the figure will be marked as "fixed" above. If NAAS is unable to fix the files, a red "failed" label will appear above. When NAAS has confirmed that the figure files meet our requirements, please download the file via the download option, and include these NAAS processed figure files when submitting your revised manuscript.

**Reproducibility:**

To enhance the reproducibility of your results, we recommend that authors of applicable studies deposit laboratory protocols in protocols.io, where a protocol can be assigned its own identifier (DOI) such that it can be cited independently in the future. Additionally, PLOS ONE offers an option to publish peer-reviewed clinical study protocols. Read more information on sharing protocols at https://plos.org/protocols?utm_medium=editorial-email&utm_source=authorletters&utm_campaign=protocols

# Author comment: Author response to Decision Letter 1

**DOI:** 10.1371/journal.pgen.1012203.r002

27 May 2026

Attachment Submitted filename: PlosGenetics_response_to_reviewers.pdf

# Peer review: Decision Letter 1

**DOI:** 10.1371/journal.pgen.1012203.r003

**Authors:** Yanshan Fang, Fengwei Yu

4 Jun 2026

Dear Dr Ryoo,

We are pleased to inform you that your manuscript entitled "The regulation of Xrp1 expression by uORFs and main ORF sequences and its function in Drosophila disease models" has been editorially accepted for publication in PLOS Genetics. Congratulations!

Before your submission can be formally accepted and sent to production you will need to complete our formatting changes, which you will receive in a follow up email. Please be aware that it may take several days for you to receive this email; during this time no action is required by you. Please note: the accept date on your published article will reflect the date of this provisional acceptance, but your manuscript will not be scheduled for publication until the required changes have been made.

Once your paper is formally accepted, an uncorrected proof of your manuscript will be published online ahead of the final version, unless you’ve already opted out via the online submission form. If, for any reason, you do not want an earlier version of your manuscript published online or are unsure if you have already indicated as such, please let the journal staff know immediately at plosgenetics@plos.org.

In the meantime, please log into Editorial Manager at https://www.editorialmanager.com/pgenetics/, click the "Update My Information" link at the top of the page, and update your user information to ensure an efficient production and billing process. Note that PLOS requires an ORCID iD for all corresponding authors. Therefore, please ensure that you have an ORCID iD and that it is validated in Editorial Manager. To do this, go to ‘Update my Information’ (in the upper left-hand corner of the main menu), and click on the Fetch/Validate link next to the ORCID field.  This will take you to the ORCID site and allow you to create a new iD or authenticate a pre-existing iD in Editorial Manager.

If you have a press-related query, or would like to know about making your underlying data available (as you will be aware, this is required for publication), please see the end of this email. If your institution or institutions have a press office, please notify them about your upcoming article at this point, to enable them to help maximise its impact. Inform journal staff as soon as possible if you are preparing a press release for your article and need a publication date.

Thank you again for supporting open-access publishing; we are looking forward to publishing your work in PLOS Genetics!

Yours sincerely,

Yanshan Fang, Ph.D., M.D.

Academic Editor

PLOS Genetics

Fengwei Yu

Section Editor

PLOS Genetics

Aimée Dudley

Editor-in-Chief

PLOS Genetics

Anne Goriely

Editor-in-Chief

PLOS Genetics

www.plosgenetics.org

BlueSky: @plos.bsky.social

----------------------------------------------------

Comments from the reviewers (if applicable):

Reviewer's Responses to Questions

**Comments to the Authors:**

**Please note here if the review is uploaded as an attachment.**

Reviewer #1: This is an improved manuscript, and quite interesting. In particular, the different regulation in minute vs retina appears to have temporal impacts - to regulate splicing in the Minute situation, you need transcription. But for the retinal model, it is translational control.

Reviewer #2: The authors appropriately addressed all concerns that had been raised by the reviewers.

I do not see any remaining problems.

Reviewer #3: The authors have addressed my concerns.

**********

**Have all data underlying the figures and results presented in the manuscript been provided?**

Large-scale datasets should be made available via a public repository as described in the *PLOS Genetics*    data availability policy, and numerical data that underlies graphs or summary statistics should be provided in spreadsheet form as supporting information.

Reviewer #1: Yes

Reviewer #2: Yes

Reviewer #3: Yes

----------------------------------------------------

**Data Deposition**

If you have submitted a Research Article or Front Matter that has associated data that are not suitable for deposition in a subject-specific public repository (such as GenBank or ArrayExpress), one way to make that data available is to deposit it in the Dryad Digital Repository. As you may recall, we ask all authors to agree to make data available; this is one way to achieve that. A full list of recommended repositories can be found on our website.

The following link will take you to the Dryad record for your article, so you won't have to re‐enter its bibliographic information, and can upload your files directly:

http://datadryad.org/submit?journalID=pgenetics&manu=PGENETICS-D-26-00363R1

More information about depositing data in Dryad is available at http://www.datadryad.org/depositing. If you experience any difficulties in submitting your data, please contact help@datadryad.org for support.

Additionally, please be aware that our data availability policy requires that all numerical data underlying display items are included with the submission, and you will need to provide this before we can formally accept your manuscript, if not already present.

----------------------------------------------------

**Press Queries**

If you or your institution will be preparing press materials for this manuscript, or if you need to know your paper's publication date for media purposes, please inform the journal staff as soon as possible so that your submission can be scheduled accordingly. Your manuscript will remain under a strict press embargo until the publication date and time. This means an early version of your manuscript will not be published ahead of your final version. PLOS Genetics may also choose to issue a press release for your article. If there's anything the journal should know or you'd like more information, please get in touch via plosgenetics@plos.org.

# Editor report: Acceptance letter

**DOI:** 10.1371/journal.pgen.1012203.r004

**Authors:** Yanshan Fang, Fengwei Yu

PGENETICS-D-26-00363R1

The regulation of Xrp1 expression by uORFs and main ORF sequences and its function in Drosophila disease models

Dear Dr Ryoo,

We are pleased to inform you that your manuscript entitled "The regulation of Xrp1 expression by uORFs and main ORF sequences and its function in Drosophila disease models" has been formally accepted for publication in PLOS Genetics! Your manuscript is now with our production department and you will be notified of the publication date in due course.

The corresponding author will soon be receiving a typeset proof for review, to ensure errors have not been introduced during production. Please review the PDF proof of your manuscript carefully, as this is the last chance to correct any errors. Please note that major changes, or those which affect the scientific understanding of the work, will likely cause delays to the publication date of your manuscript.

Soon after your final files are uploaded, unless you have opted out or your manuscript is a front-matter piece, the early version of your manuscript will be published online. The date of the early version will be your article's publication date. The final article will be published to the same URL, and all versions of the paper will be accessible to readers.

For Research Articles, you will receive an invoice from PLOS for your publication fee after your manuscript has reached the completed accept phase. If you receive an email requesting payment before acceptance or for any other service, this may be a phishing scheme. Learn how to identify phishing emails and protect your accounts at https://explore.plos.org/phishing.

Thank you again for supporting PLOS Genetics and open-access publishing. We are looking forward to publishing your work!

With kind regards,

Anita Estes

PLOS Genetics

On behalf of:

The PLOS Genetics Team

Carlyle House, Carlyle Road, Cambridge CB4 3DN | United Kingdom

plosgenetics@plos.org | +44 (0) 1223-442823

plosgenetics.org | Twitter: @PLOSGenetics
